# Cholecystohepatic duct detected during laparoscopic cholecystectomy: a case report

**DOI:** 10.1186/s40792-020-0786-3

**Published:** 2020-01-13

**Authors:** Koki Maeda, Masami Tabata, Tatsuya Sakamoto, Yu Fujimura, Taijiro Takeuchi, Ryosuke Desaki, Motoyuki Kobayashi, Ichiro Ohsawa, Kenji Kato, Makoto Iwata, Takayuki Sanda

**Affiliations:** 1 Department of Hepatobiliary-Pancreatic and Transplant Surgery, Mie University School of Medicine, 2-174 Edobashi, Tsu, Mie 514-0001 Japan; 2 Department of Surgery, Matsusaka Central General Hospital, 102 Kobo, Kawaimachi, Matsusaka, Mie 515-8566 Japan

**Keywords:** Cholecystohepatic duct, Aberrant hepatic duct, Laparoscopic cholecystectomy

## Abstract

**Background:**

The cholecystohepatic duct is a rare form of an aberrant hepatic duct that connects to the gallbladder. Although cholecystohepatic duct is reported to be a very rare anomaly, injury of cholecystohepatic duct during cholecystectomy may result in serious complications. Herein, we present a case of cholecystohepatic duct in the ventral branch of the right posterior inferior segmental bile duct detected during laparoscopic cholecystectomy.

**Case presentation:**

A 77-year-old woman with cholecystolithiasis had been referred to our hospital for surgery. Drip infusion cholecystocholangiography-computed tomography revealed a bile duct branch without communication between the intra- and extrabiliary systems, although the existence of this aberrant hepatic duct was not suspected preoperatively. A 4-port laparoscopic cholecystectomy was performed. After critical view of safety was confirmed, the cystic artery and duct were divided after double clipping. During antegrade mobilization of the gallbladder from the gallbladder bed, a thin, white cord-like material connecting the gallbladder neck and bed was detected. After clipping and dividing it, a cholecystohepatic duct injury was recognized through rechecking the results of the preoperative examinations. Biliary reconstruction was considered unnecessary because of the lesion’s small drainage area. The postoperative course was uneventful, and an enhanced computed tomography performed 6 months after the surgery revealed a dilation in the ventral branch of the right posterior inferior segmental bile duct. The patient’s liver function remained normal, and she had no symptoms of cholangitis 42 months after the surgery.

**Conclusions:**

Although cholecystohepatic duct is a rare anomaly compared to other aberrant hepatic ducts, surgeons performing cholecystectomy should always keep its existence in mind to avoid serious postoperative complications. Ideally, preoperative detection of cholecystohepatic duct is preferable, but even if it is detected during surgery, the appropriate management according to the drainage area is also important.

## Background

Aberrant hepatic ducts are defined as bile duct branches that assume the biliary drainage of a sector, segment, or limited area of the liver, connecting directly to the extrahepatic bile duct, cystic duct, or gallbladder. Cholecystohepatic duct (CHD) is a specific form of the aberrant hepatic duct connecting to the gallbladder [1]. Although CHD is reported to be a very rare anomaly, with a frequency of 0.7–1.2% [2, 3], injury to CHD during cholecystectomy may result in various complications such as bile leakage, cholangitis and liver abscess. Here, we present the case of a patient with CHD in the ventral branch of the right posterior inferior segmental bile duct (B6), detected and injured during laparoscopic cholecystectomy. The present case report demonstrates one of the feasible management of a small CHD injury.

## Case presentation

**Fig. 1:**
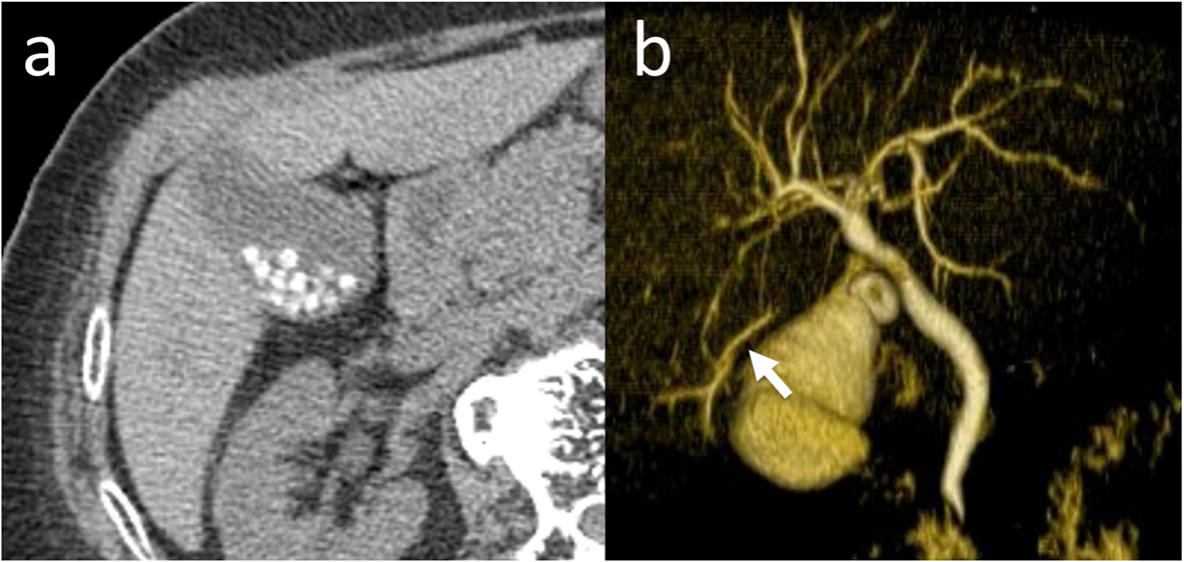
**a** Computed tomography scan revealing multiple calcified stones in the gallbladder. **b** Preoperative drip infusion cholecystocholangiography-computed tomography showing a bile duct branch with no communication between the intra- and extrabiliary systems (arrow)

A 77-year-old female patient had been followed up at a nearby hospital for asymptomatic cholecystolithiasis for the past 10 years. She was referred to our hospital for surgery after an abdominal ultrasound revealed an increase in the number of gallstones. There seemed to be no operative indication for asymptomatic cholecystolithiasis, but she requested cholecystectomy. Although laboratory data showed no abnormalities, the CT scan revealed multiple calcified stones in the gallbladder, measuring about 4 mm in maximum diameter (Fig. 1a). Drip infusion cholecystocholangiography-computed tomography (DIC-CT) revealed that the cystic duct was connected to the upper third of the extrahepatic bile duct. In the cranial side of the gallbladder, the bile duct branch presented no communication between the intra- and extrabiliary systems (Fig. 1b). However, the existence of this aberrant hepatic duct was not suspected preoperatively. Thus, further biliary examination such as magnetic resonance cholangiopancreatography (MRCP) and endoscopic ultrasonography (EUS) had not been performed.

**Fig. 2:**
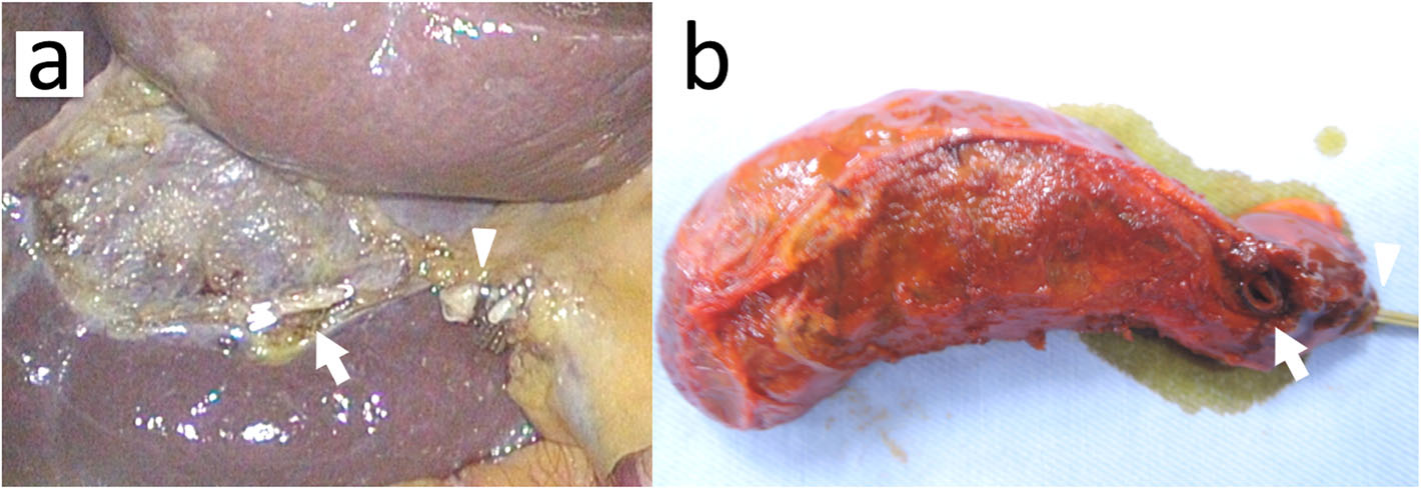
**a** Intraoperative photographs revealing the cholecystohepatic duct stump in the gallbladder bed (arrow) and the stump of the cystic duct (arrowhead). **b** The cholecystohepatic duct orifice was confirmed in the resected gallbladder neck (arrow). An arrowhead shows the cystic duct stump

A 4-port laparoscopic cholecystectomy was performed. Laparoscopy revealed that the gallbladder had no inflammatory changes. After a critical view of safety was confirmed, the cystic artery and duct were divided after double clipping. During antegrade mobilization of the gallbladder from the gallbladder bed, a thin, white cord-like material connecting the gallbladder neck and bed was detected. Assuming that this cord consisted of a cystic vein, we divided it after clipping. However, yellowish bile on the cut end of the cord (Fig. 2a) indicated a bile duct injury. Although we had considered performing intraoperative cholangiography, it could not be completed due to technical difficulty. On rechecking the DIC-CT in detail, we concluded that the cut bile duct was the aberrant ventral branch of B6, which was connected to the gallbladder neck. The drainage area of this aberrant hepatic duct was small because the dorsal and lateral branches of B6 had no anomalies. Therefore, we had finished the surgery without biliary reconstruction.

The cut end of the CHD was observed in the gallbladder bed of the resected gallbladder (Fig. 2b). Preliminary pathological report indicated that the CHD had a normal bile duct structure. The patient was discharged 5 days after the surgery without any complications.

**Fig. 3:**
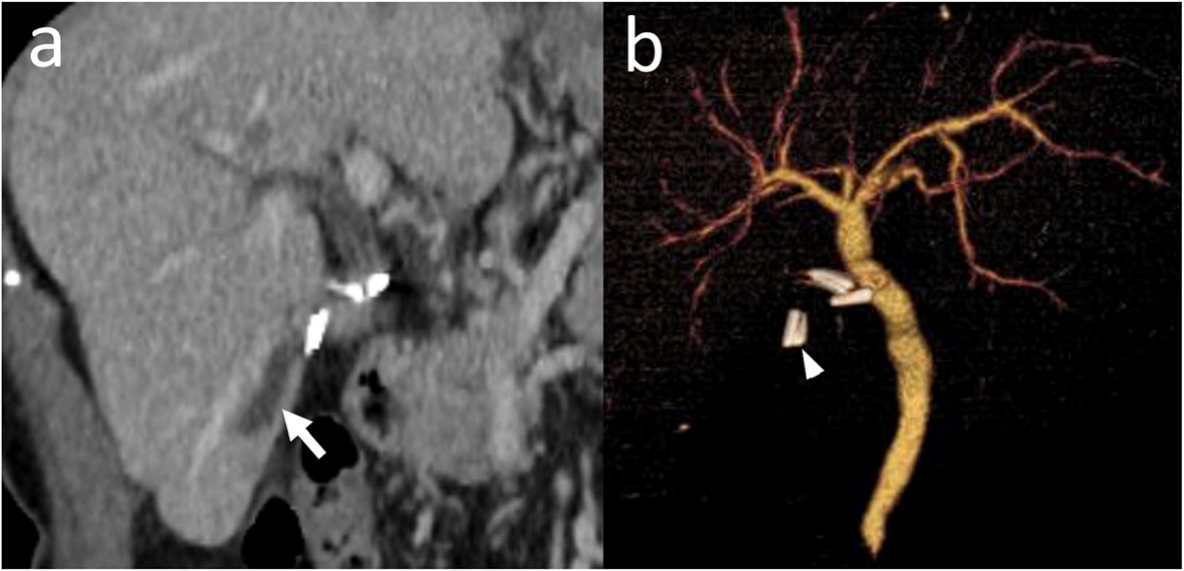
**a** The computed tomography scan performed 6 months after the surgery reveals the dilatation of the ventral branch of right posterior inferior segmental bile duct (arrow). **b** Drip infusion cholecystocholangiography-computed tomography performed on postoperative day 11 reveals the clip on the cholecystohepatic duct (arrowhead) just below the cystic duct clips

The DIC-CT performed on postoperative day 11 revealed the clip on the CHD just below the cystic duct clips (Fig. 3b). An enhanced CT scan performed after 6 months post-surgery revealed the ventral branch dilation in B6 (Fig. 3a). The patient’s liver function remained normal and she had no symptoms of cholangitis 42 months after the surgery.

## Discussion

CHD was first reported by Neuhof et al. [1] in 1945 as an anomaly that caused the hepatic ducts to drain a certain area of the liver into the gallbladder. Some authors use the term of “Cystohepatic duct” for this anomaly [4], but the cystohepatic duct is defined as those draining variable portion of the right lobe into the cystic duct [5]. Thus, CHD is considered as optimal terminology for this case. CHD is thought to arise as a result of abnormal biliary tract embryology, and its incidence is much lower than that of other aberrant hepatic ducts. A large case series based on operative and cholangiographic findings demonstrated an overall incidence of CHD as 0.07% (only 1 case in a total of 1410 cholecystectomies) [4, 5].

As a cause of bile duct injury during cholecystectomy, anatomical anomalies of the bile duct are common, as well as a surgical difficulty due to severe inflammation and adhesion. Anomalous drainage of the right posterior hepatic duct joining into the common hepatic duct is the most frequent cause of bile duct injury [6–9]. However, several cases of bile duct injury have been reported in CHD [10–13]. In all these cases, CHD was not recognized either pre- or intraoperatively, but was confirmed postoperatively because of bile leakage. Thus, preoperative detection of CHD seems to be difficult but important for avoiding a bile duct injury. We routinely perform MRCP or DIC-CT before cholecystectomy to evaluate biliary anomaly. MRCP is preferable to avoid radiation exposure but it is difficult to perform rapidly unless emergency in our hospital. Thus, we often perform DIC-CT as in this case. In the present case, the preoperative DIC-CT revealed the CHD as a small bile duct branch with no communication between intra- and extrabiliary systems. However, it was missed due to a lack of awareness of the surgeons. If we had been experienced such a case before, we might be noticed it. Therefore, we consider that the most important tips to detect a CHD is to recognize and suspect its existence. When a bile duct branch with unclear confluence near the gallbladder was described by preoperative image, we should suspect it as a CHD.

To avoid bile duct injury, as well as preoperative detection of bile duct anomalies, safety surgical procedure is extremely important. The critical view of safety has been established as a safety procedure of laparoscopic cholecystectomy [14], and also the dissection by exposing the inner layer of the subserosal layer has been known as an effective surgical procedure [15]. However, differ from the aberrant hepatic ducts joining into the extrahepatic bile duct, even if CHD was detected before surgery and safety surgical procedures were completely performed, it must be divided in order to resect the gallbladder. Three surgical options are available for managing CHD that is detected before or during cholecystectomy. First, in case the drainage area of CHD is large, biliary reconstruction is necessary. Kurata et al. [16] reported reconstruction of posterior sectional bile duct using Roux-en-Y choledochojejunostomy and Hamada et al. [17] described an anastomosis of the anterior inferior segmental bile duct to the remnant cystic duct. Secondly, if the drainage area of CHD is small, closure of CHD is permissible. As the initial management of a bile duct injury, Longmire et al. [18] described the ligation of small segmental ducts (i.e., < 1 mm), and recommended bile duct reconstruction for ducts with a diameter of ≥ 2 mm. In our presenting case, the CHD was rather thin with a drainage area comprising only a part of the right posterior inferior segment. Therefore, we considered that biliary reconstruction was unnecessary. No clinical problems were observed over 4 years following the surgery. However, dilatation of the ligated CHD remains without liver atrophy, requiring a long-term follow-up, focused on the elevation of liver enzymes and the occurrence of obstructive cholangitis. Thirdly, the remaining surgical option is CHD preservation. In cases where the CHD connects to the cystic duct (i.e., cystohepatic duct) or the neck of the gallbladder, it is possible to preserve it through gallbladder dissection on the right side of the CHD confluence (i.e., subtotal cholecystectomy). Kurata et al. [16] and Hirai et al. [19] reported the case of subtotal cholecystectomy, preserving the CHD or cystohepatic duct.

When the CHD was noticed after surgery by bile leakage from the drain or bile peritonitis, strategy for the management of resected CHD had not been established. Rathore et al. [11] experienced a case of bile leakage from the drain on a postoperative day 1. The CHD injury was confirmed by drainage tubography on postoperative day 11 and the bile leakage stopped 27 days after the operation. Uemura et al. [13] reported that bile leakage from the resected posterior sectional bile duct improved following percutaneous abdominal drainage, without percutaneous transhepatic biliary drainage or reoperation. However, Zrin et al. [20] performed a reoperation and ligated the CHD for bile peritonitis.

## Conclusions

Although CHD is a rare anomaly compared to other aberrant hepatic ducts, surgeons performing cholecystectomy should always keep its existence in mind to avoid serious postoperative complications. Ideally, preoperative detection of CHD is preferable, but even if it is detected during surgery, the appropriate management according to the drainage area is also important.

## Abbreviations

CHD: Cholecystohepatic duct
DIC-CT: Drip infusion cholecystocholangiography-computed tomography
EUS: Endoscopic ultrasonography
MRCP: Magnetic resonance cholangiopancreatography
